# Origin and evolution of a gibberellin‐deactivating enzyme GAMT

**DOI:** 10.1002/pld3.287

**Published:** 2020-12-25

**Authors:** Chi Zhang, Minta Chaiprasongsuk, Andre S. Chanderbali, Xinlu Chen, Jianyu Fu, Douglas E. Soltis, Feng Chen

**Affiliations:** ^1^ Department of Plant Sciences University of Tennessee Knoxville TN USA; ^2^ Department of Botany Faculty of Science Kasetsart University Bangkok Thailand; ^3^ Department of Biology University of Florida Gainesville FL USA; ^4^ Florida Museum of Natural History University of Florida Gainesville FL USA; ^5^ Key Laboratory of Tea Quality and Safety Control Ministry of Agriculture and Rural Affairs Tea Research Institute Chinese Academy of Agricultural Sciences Hangzhou China

**Keywords:** gene loss, plant hormone, reproduction, seed plants

## Abstract

Gibberellins (GAs) are a major class of plant hormones that regulates diverse developmental programs. Both acquiring abilities to synthesize GAs and evolving divergent GA receptors have been demonstrated to play critical roles in the evolution of land plants. In contrast, little is understood regarding the role of GA‐inactivating mechanisms in plant evolution. Here we report on the origin and evolution of GA methyltransferases (GAMTs), enzymes that deactivate GAs by converting bioactive GAs to inactive GA methylesters. Prior to this study, *GAMT* genes, which belong to the SABATH family, were known only from *Arabidopsis*. Through systematic searches for *SABATH* genes in the genomes of 260 sequenced land plants and phylogenetic analyses, we have identified a putative *GAMT* clade specific to seed plants. We have further demonstrated that both gymnosperm and angiosperm representatives of this clade encode active methyltransferases for GA methylation, indicating that they are functional orthologs of *GAMT*. In seven selected seed plants, *GAMT* genes were mainly expressed in flowers and/or seeds, indicating a conserved biological role in reproduction. *GAMT* genes are represented by a single copy in most species, if present, but multiple copies mainly produced by whole genome duplications have been retained in Brassicaceae. Surprisingly, more than 2/3 of the 248 flowering plants examined here lack *GAMT* genes, including all species of Poales (e.g., grasses), Fabales (legumes), and the large Superasterid clade of eudicots. With these observations, we discuss the significance of *GAMT* origination, functional conservation and diversification, and frequent loss during the evolution of flowering plants.

## 1 INTRODUCTION

Gibberellins (GAs) are a class of diterpenoid plant hormones that has played an important role in land plant evolution (Zi et al., 2014). Bryophytes, the basal lineage of land plants, do not contain GAs, but some of them have been demonstrated to use GA precursors to regulate development (Hirano et al., 2007; Yasumura et al., 2007). All vascular plants, including lycophytes, ferns, gymnosperms, and angiosperms, synthesize GAs as an essential plant hormone (MacMillan, 2001). In addition to their conserved roles in regulating some fundamental development programs such as stem elongation and leaf expansion (Sun, 2008), GAs have acquired lineage‐specific functions among vascular plants. In seed plants (gymnosperms and angiosperms), GAs promote seed germination (Urbanova & Leubner‐Metzger, 2016). In angiosperms, GAs regulate flowering (Blazquez et al., 1998). Such lineage/developmental program‐specific functions of GAs may have played an important role in the diversification of vascular plants and their adaptations. Thus, it is of fundamental interest to ask how GAs achieve such lineage/developmental program‐specific functions.

For biosynthesis of GAs, three types of genes are involved: terpene synthases, cytochrome P450 monooxygenases, and 2‐oxoglutarate‐dependent dioxygenases (Yamaguchi, 2008). The inability to synthesize GAs by the moss *Physcomitrella patens* has been partly attributed to the lack of one key P450 gene of the CYP88 family (Rensing et al., 2008). Therefore, evolving the complete set of the three types of genes is essential to enable GA biosynthesis in vascular plants. Recent studies have shown the importance of evolution of GA perception in defining specific functions of GAs. GID1, the receptor of GAs, evolved from carboxylesterase in ancestral vascular plants after the split from the bryophyte lineage (Ueguchi‐Tanaka et al., 2007; Yoshida et al., 2018). The lycophyte GID1s have been termed initial GID1s because of their inferior affinity toward bioactive GAs than those of GID1s in seed plants. The fern GID1s have been called adapted GID1s, which exhibit improved adjustments for binding different GAs. The seed plant GID1s have been diversified. For instance, nearly all eudicots contain two types of GID1, named A‐ and B‐type, with the latter type associated with organ‐specific functions (Griffiths et al., 2006; Yoshida et al., 2018). Besides biosynthesis and perception, inactivation of GAs also plays a role in regulating GA activities (Hedden & Phillips, 2000; Olszewski et al., 2002), for which multiple mechanisms are known to exist. These include 2β‐hydroxylation catalyzed by GA 2‐oxidases (Thomas et al., 1999), conjugation to form glucosyl esters and glucosides (Schneider et al., 1992), epoxidation catalyzed by a cytochrome P450 monooxygenase (Zhu et al., 2006) and methylation of the carboxyl group catalyzed by GA methyltransferase (GAMT) to form GA methylesters (Varbanova et al., 2007). Little is understood on the role of GA inactivation in plant evolution.

GAMT‐catalyzed deactivation of GAs is the most recently discovered mechanism of GA inactivation (Varbanova et al., 2007). The model plant *Arabidopsis* contain two *GAMT* genes designated *AtGAMT1* and *AtGAMT2*. Both *AtGAMT1* and *AtGAMT2* showed the highest levels of expression during seed development (Varbanova et al., 2007). Using overexpression and knockout lines, the function of *AtGAMTs* in *Arabidopsis* was demonstrated to be deactivating bioactive GAs during seed development (Varbanova et al., 2007). Transgenic tobacco, petunia, and tomato plants overexpressing *Arabidopsis GAMT*s exhibit the phenotypes of GA deficit (Nir et al., 2014; Varbanova et al., 2007), supporting the role of GAMT in GA catabolism. GAMTs belong to the methyltransferase family called SABATH (D'Auria et al., 2003). Other known members of the SABATH family that methylate phytohormones include indole‐3‐acetic acid methyltransferase (IAMT) (Qin et al., 2005; Zhao et al., 2007), salicylic acid methyltransferase (SAMT) (Chen et al., 2003; Ross et al., 1999), and jasmonic acid methyltransferase (JAMT) (Seo et al., 2001). *IAMT* has been demonstrated to be ancient and conserved in seed plants (Zhao et al., 2008), while *SAMT* and *JAMT* appear to have arisen multiple times during the evolution of seed plants (Chaiprasongsuk et al., 2018). Despite discovery in *Arabidopsis* more than a decade ago (Varbanova et al., 2007), the origin, evolution, and function of *GAMT* genes in other plants is completely unknown. In this study, we use a comparative genomics approach to identify putative *GAMT* genes, and investigate their origin and evolution in the context of land plant evolution.

## 2 METHODS

### 2.1 Sequence retrieval and analysis

All protein models of the 260 sequenced plant genomes were downloaded from Phytozome v12.1 (https://phytozome.jgi.doe.gov/pz/portal.html), *Brassica* Database (http://brassicadb.org/brad/index.php), *Citrus* Genome Database (CGD, https://www.citrusgenomedb.org), Cucurbit Genomics Database (CuGenDB, http://cucurbitgenomics.org), Hardwood Genomics Project (HWG, https://www.hardwoodgenomics.org) or respective databases referred in literatures (Table S1). This dataset was searched for SABATH proteins by HMM search with E‐value of 1e‐5 against the HMM profile Methyltransf_7 (PF03492) (Finn et al., 2016). To identify and categorize GA2ox proteins, a method was applied based on two rounds of HMM searches (Johnson et al., 2010). An HMM‐based in‐house script was first used to identify proteins that contain both DIOX_N (PF14226) and 2OG‐FeII_Oxy (PF03171) conserved domains. Next, two HMM profiles, one for C19‐GA2ox (C19G) and the other for C20‐GA2ox (C20G), were made with specific conserved domains of GA2ox proteins from selected plant species (Table S4) as previously reported (Huang et al., 2015). Lastly, individual GA2ox proteins were separated into the C19‐GA2ox group and the C20‐GA2ox group by being subjected to HMM search against C19G and C20G HMM profiles with an E‐value of 1e‐5.

### 2.2 Phylogenetic reconstruction

All newly identified SABATH methyltransferases with a minimum length of 250 amino acids were used for phylogenetic reconstruction. Multiple protein sequence alignments were made with MAFFT version 7.369b under L‐INS‐I strategy (Katoh & Standley, 2013). The phylogenetic tree was generated by RAxML v8.2 using the LG + G+F model with 1,000 bootstraps (Stamatakis, 2014). The phylogenetic tree based on plant taxonomy was constructed using phyloT (https://phylot.biobyte.de). All phylogenetic trees were visualized by iTOL (Letunic & Bork, 2016).

### 2.3 Gene cloning, protein expression, and enzyme assays

Full‐length cDNAs for two *GAMT* genes from *Ginkgo biloba*, three *GAMT* genes from *Brassica rapa* and 11 *SABATH* genes from *Brachypodium distichton* were cloned from respective plant tissues by RT‐PCR with primers(Tabel S5) as previously described (Zhao et al., 2008). Putative full‐length cDNAs for all other GAMT or SABATH genes analyzed in this study were synthesized. All cDNAs were cloned into pET‐32a vector (MilliporeSigma) and confirmed by sequencing. Proteins were expressed in the *Escherichia coli* strain BL21 (DE3) (Stratagene) then tested for methyltransferase activities using radiochemical assays. Each assay was performed with a 50 μL volume containing 50 mM Tris‐HCl, pH 8.0, 1mM substrates, 3 μL ^14^C‐*S*‐adenosyl‐L‐methionine (SAM) (PerkinElmer), and 1 μL purified enzyme. After incubation at 30°C for 30 min, the assays were extracted with 150 μL ethyl acetate. The organic phase was counted in a scintillation counter (Beckman Coulter) to measure the relative methyltransferase activity.

### 2.4 Gene expression data retrieval

The gene expression data for *Ginkgo biloba* were retrieved from http://gigadb.org/dataset/100209 (Guan et al., 2016). The gene expression data for *Camelina sativa* and *Vitis vinifera* were analyzed through http://bar.utoronto.ca/ (Fucile et al., 2011). The gene expression data for *Picea abies* were retrieved from http://congenie.org (Sundell et al., 2015). The gene expression data for *Phalaenopsis equestris* were retrieved from http://orchidstra2.abrc.sinica.edu.tw (Chao et al., 2017). The gene expression data for *Musa acuminata* were retrieved from https://banana‐genome‐hub.southgreen.fr (Droc et al., 2013). The gene expression data for *Citrus sinensis* were retrieved from http://citrus.hzau.edu.cn (Wang et al., 2014). Read counts, fragments per kilobase million (FPKM) values, reads per kilobase million (RPKM) values or relative expression values were acquired via gene id search or blast search with putative GAMTs of that species in each database. Tissue specific expression data were later entered into tables, standardized to relative expression values by dividing highest expression value in each group and applied to drawing histograms in Excel, respectively. Standard deviations were marked if such information is available from that database.

## 3 RESULTS AND DISCUSSION

### 3.1 Comparative analysis of the SABATH family in 260 sequenced land plants and the identification of a putative GAMT clade

**FIGURE 1:**
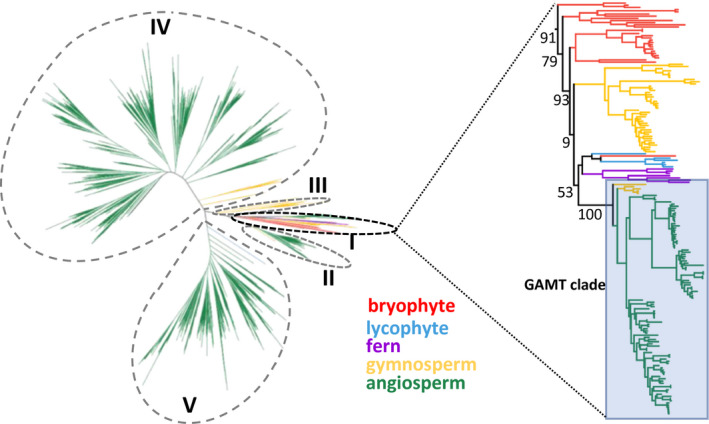
Phylogenetic analysis of SABATH proteins from 260 sequenced plants (Table S1). In this unrooted phylogenetic tree, the SABATHs were clustered into five groups I to V. Group I was enlarged to illustrate individual plant lineages with bootstrap values (percent out of 1,000 iterations) shown. The shaded clade indicates the putative GAMT clade

We compiled a total of 260 land plants with sequenced genomes, including 248 species of angiosperms, six species of gymnosperms, two species of ferns, one species of lycophyte and three species of bryophytes, from various public sources (Table S1). Then, the complete proteome for each of the 260 sequenced land plants was downloaded to a local server and the entire dataset was searched for SABATH proteins. A total of 6,458 SABATH proteins was identified with an average of 25 proteins per plant genome. The sizes of the SABATH family ranged from 1 (*Apostasia shenzhenica* and *Pogostemon cablin*) to 115 (*Triticum aestivum*). Next, the SABATH proteins were subject to phylogenetic analysis. SABATHs from seed plants were placed into five groups (I to V) (Figure 1). Group I contains SABATHs from all major lineages of land plants bryophytes, lycophytes, ferns, gymnosperms and angiosperms. *Arabidopsis* GAMT1 and GAMT2, the only two known GAMTs, belong to group I. Group II is specific to seed plants. It is noteworthy that all IAMTs that have been functionally characterized, including those from the angiosperms *Arabidopsis*, rice and poplar and the gymnosperm spruce, belong to group II. Group III is specific to gymnosperms. Group IV contains SABATHs from both gymnosperms and angiosperms. In contrast, group V is specific to angiosperms. Within group I, the SABATHs from angiosperms including the two *Arabidopsis* GAMTs and a subset of the SABATHs from gymnosperms form a clade with strong bootstrap support (100%) (Figure 1). This was defined as the putative GAMT clade. The GAMT clade was clustered with the SABATHs from bryophytes, lycophytes, and ferns with poor bootstrap support (53%).

### 3.2 The catalytic activity of selected members in the GAMT clade

**FIGURE 2:**
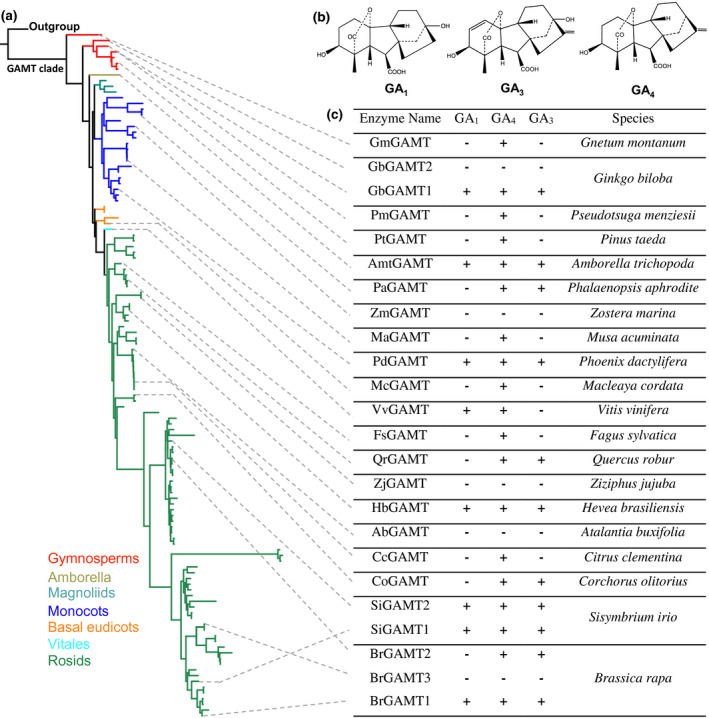
GAMT clade and biochemical activities. (a) Phylogeny of the GAMT clade with major lineages illustrated. (b) The chemical structures of gibberellin A_1_(GA_1_), gibberellin A_3_ (GA_3_), and gibberellin A_4_ (GA_4_). (c) Representative GAMTs and their activity towards to GA_1_, GA_3,_ and GA_4_. “+” and “−” indicate “active” and “inactive,” respectively

Within the putative GAMT clade, the phylogeny of the putative GAMTs (Figure 2a) is largely congruent to the species tree of seed plants established by APG IV (2016), implying that *GAMT* is conserved in seed plants. To determine whether any of the members in this putative *GAMT* clade besides the two Arabidopsis *GAMTs* encode enzymes with GAMT activity, we conducted biochemical analyses with representatives for methyltransferase activity via *in vitro* assays using gibberellin A1(GA_1_), gibberellin A_3_ (GA_3_), and gibberellin A_4_ (GA_4_) (Figure 2b), three of the most widely occurring bioactive GAs (MacMillan, 2001), as substrates. A total of 24 putative GAMTs from 20 species in the GAMT clade (Figure 2c) was selected for enzyme assays. A full‐length cDNA for each of the 24 *GAMT* genes was expressed in *Escherichia coli* and the recombinant protein tested for methyltransferase activity in *in intro* assays. Nineteen of the 24 proteins showed activity with GA_4._ Eight and eleven of the 19 active SABATHs also had catalytic activity with GA_1_ and GA_3_ as a substrate, respectively (Figure 2c). None of the 19 proteins with GAMT activity showed activity with IAA, JA, or SA as substrates, indicating that these GAMTs have strict substrate specificity towards GAs.

### 3.3 Expressed patterns of *GAMT* genes in selected seed plants

**FIGURE 3:**
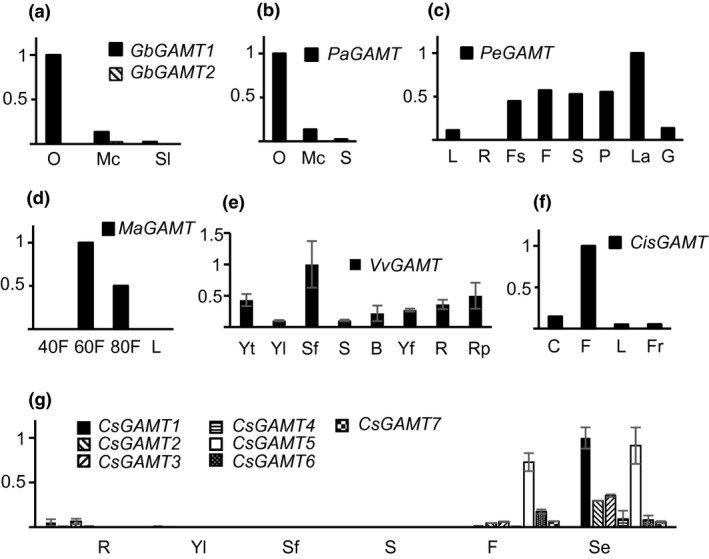
Expression patterns of *GAMT* genes in selected species based on their expression data in public sources. (a) *GbGAMT1* and *GbGAMT2* from *Ginkgo biloba*; (b) *PaGAMT* from *Picea abies*; (c) *PeGAMT* from *Phalaenopsis equestris*; (d) *MaGAMT* from *Musa acuminata*; (e) *VvGAMT* from *Vitis vinifera*; (f) *CisGAMT* from *Citrus sinensis*; (g) *CsGAMT1* to *CsGAMT7* from *Camelina sativa*. O, ovules; Mc, male cones; Sl, dtem and leaves; S, stem; L, leaf; R, root; Fs, floral stalk; F, flower; S, sepal; P, petal; La, labellum; G, gynostemium; 40F, 40‐day‐fruit; 60F, 60‐day‐fruit; 80F, 80‐day‐fruit; Yt, young tendril; Yl, young leaf; Sf, senescent leaf; B, bud; Yf, young flower; Rp, ripening pericarp; C, callus; Fr, fruit; Ro, rosette; Cl, cauline leaf; Se, seed. The highest level of expression in each species was arbitrarily set at 1.0. Standard deviations were marked with error bars in (e) and (g), not in other figures due to lack of such information

To gain insight into the biological processes in which *GAMT* genes may be involved in seed plants we examined their expression patterns in seven species representing gymnosperms (*Ginkgo biloba*, *Picea abies*) and angiosperms, including monocots (*Phalaenopsis equestris*, *Musa acuminate*) and eudicots (*Vitis vinifera*, *Citrus sinensis*, and *Camelina sativa*) using public expression databases (Figure 3). In *G. biloba*, only one of two putative GAMTs showed activity with GAs and its *bona fide GAMT* gene expressed mainly in ovules (Figure 3a). The similar expression pattern was observed in another gymnosperm *P. abies* (Figure 3b) In *P. equestris*, *GAMT* was mainly expressed in the flower, especially in the labellum (Figure 3c). In *M. acuminata*, *GAMT* expression was observed in the fruit, with higher transcript levels detected during ripening (Figure 3d). In grapevine, its *GAMT* gene showed highest level of expression in senesced leaves. It also showed expression in young flowers, roots and pericarp (Figure 3e). In *C. sinensis*, *GAMT* was mainly expressed in flowers (Figure 3f). There are seven *GAMT* genes in the *C. sativa* genome; all copies were expressed mainly during reproductive growth, with five showing the highest level of expression in early or early‐mid stages of seed development; the other three gene copies showed the highest levels of expression in flowers (Figure 3g).

### 3.4 Retention after duplication of *GAMT* genes in Brassicaceae

**FIGURE 4:**
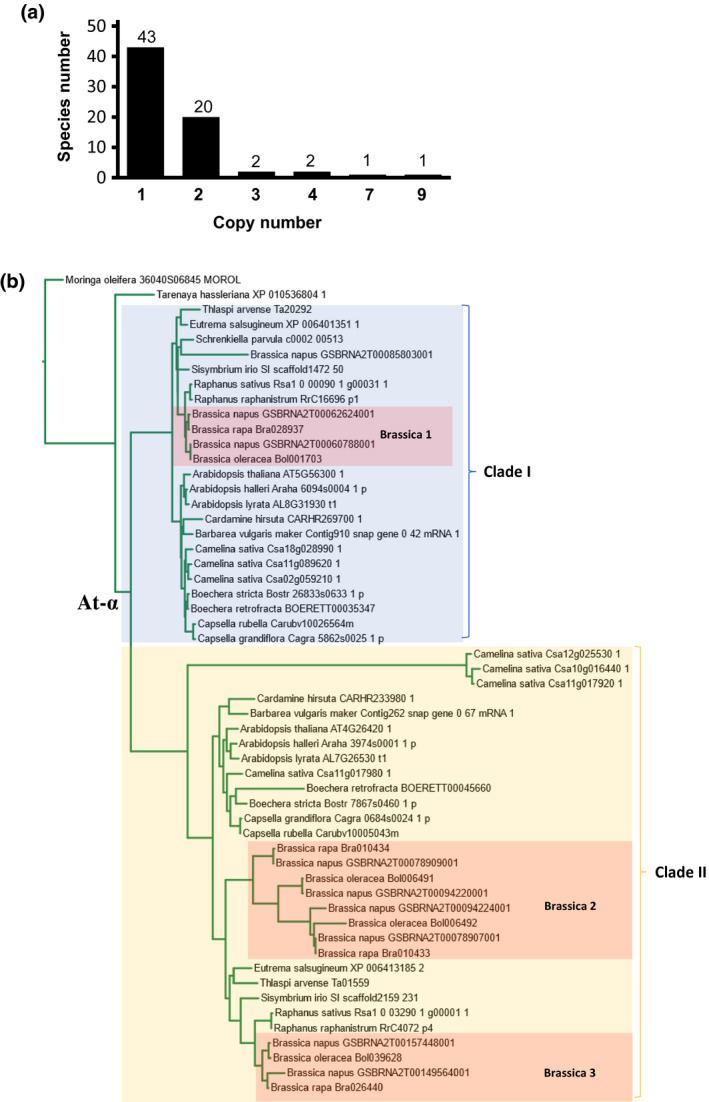
Copy number of *GAMT* and its duplication in Brassicaceae. (a) Distribution of copy numbers of *GAMT* genes among 69 *GAMT*‐containing flowering plants. (b) Phylogenetic tree of GAMTs in Brassicales. The two clades (clade I and clade II) in blue and in yellow, respectively, depict two clades that resulted from a whole gene duplication event occurred in the ancestor of Brassicaceae known as At‐α. The three smaller blocks (Brassica 1, Brassica 2, and Brassica 3) for Brassica GAMTs indicate a possible outcome of whole genome triplication event

Among the 69 flowering plants that contains *GAMT* genes, about a third (26 species) contains more than one copy of *GAMT* gene (Figure 4a). It is interesting to note most of the 26 species with two or more copies of *GAMT* belong to Brassicaceae. In fact, 18 of the 19 species of Brassicaceae, except *Schrenkiella parvula*, that were analyzed in this study contain two or more copies of *GAMT* genes (Figure 4b). Fourteen species, including *Arabidopsis*, contain two *GAMT* genes. In contrast, *Brassica rapa*, *B. oleracea*, *B. napus,* and *Camelina sativa* contain 4, 4, 9, and 7 *GAMT* genes, respectively. GAMTs of Brassicaceae forms two clades I and II (Figure 4b). Except *S. parvula*, all other 17 species contain *GAMT* in both clade I and clade II. This implies the duplication of *GAMT* in the common ancestor of Brassicaceae, most likely as an outcome of the whole genome duplication event that occurred in the common ancestor of Brassicaceae known as At‐α (Cardinal‐McTeague et al., 2016). This proposition is supported by the localization of *AtGAMT1* (At4g26420) and *AtGAMT2* (At5g56300) on two duplicated chromosomal segments. Within clade II, GAMTs from Brassica occurred in separate groups, which is likely due to a Brassica‐specific whole genome triplication event (Cheng et al., 2014). *B. napus* is a recent allopolyploid obtained by a cross between *B. oleracea* and *B. rapa* (Chalhoub et al., 2014). Consistent with this evolutionary history, each orthologus pair of GAMTs has one copy in *B. oleracea*, one copy in *B. rapa* and two copies in *B. napus* (Figure 4b). Notably, one of the two groups of Brassica GAMTs in clade II (Brassica 2) contains GAMTs from *B. oleracea*, *B. rapa,* and *B. napus* all as tandem repeats (Figure 4b), indicating that tandem duplication contributed to the expansion of the GAMT family in Brassica, although not to other Brassicaceae. Similarly, the three‐*GAMT* clusters of *C. sativa* within clade I and clade II were also likely an outcome of whole genome triplication (Kagale et al., 2014). Genome duplication is common in land plant evolution (Panchy et al., 2016; Qiao et al., 2019); it is one important mechanism leading to gene duplication and functional divergence. The doubling or further amplification of *GAMT* genes in Brassicaceae suggests that some of the Brassicaceae *GAMT* genes may have acquired specific specificity towards different GAs, as demonstrated for the two GAMTs in *Arabidopsis* (Varbanova et al., 2007) and the two active GAMTs in *B. rapa* (Figure 2c).

### 3.5 The apparent ortholog of *GAMT* gene is absent in about 2/3 of the 248 flowering plants

**FIGURE 5:**
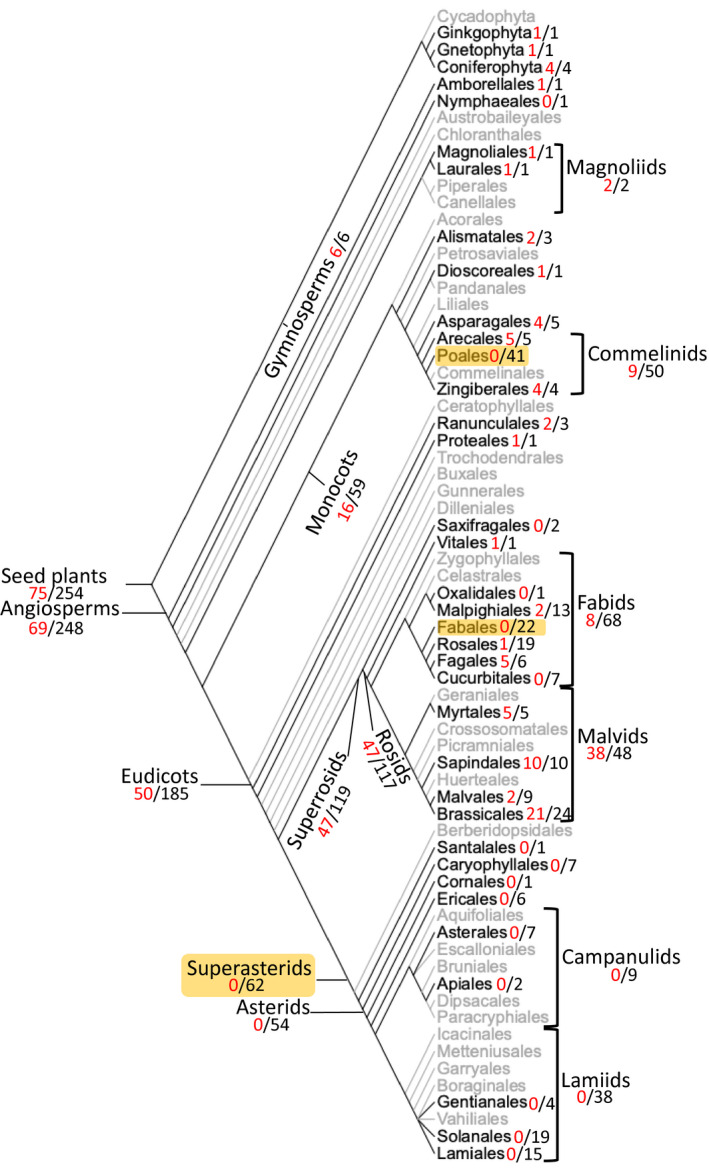
Presence/absence of *GAMT* genes in seed plants. The phylogeny was redrawn from APG IV (2016). The lineages in gray indicated that no species from those lineages was analyzed in this study. The two numbers (red and black) represent the number of species containing the *GAMT* gene and the total number of species from that specific lineage analyzed in this study. Three taxa with complete loss of *GAMTs* were shaded

When the *GAMT* genes in the *GAMT* clade were mapped to individual species, all six species of gymnosperms contain *GAMT* genes. In contrast, only 69 out of the 248 flowering plants contain *GAMT* genes (Figure 5), including some species‐rich lineages. No *GAMT* gene was identified in any of the 41 species in the order Poales. Among eudicots, *GAMT* appeared to be absent in all 22 species examined in Fabales and all 62 species included here from Superasterids. The absence of *GAMT* genes in certain land plant species could be due to incomplete coverage and/or poor quality of genome sequencing. Nonetheless, their absence in certain angiosperm lineages with a large number of species having sequenced genomes (e.g., Poales, Fabales, and Superasterids) can be concluded with confidence; these absences from entire clades imply multiple independent losses during angiosperm evolution.

As described earlier, there are several known mechanisms of GA inactivation, with GA 2β‐hydroxylation catalyzed by GA 2‐oxidases (GA2ox) considered the most important mechanism (Thomas et al., 1999). There are two types of GA2ox: C19‐GA2ox using C19 GAs as substrates and C20‐GA2ox using C20 GAs as substrates. *Arabidopsis* and rice contain five and seven C19‐*GA2ox* genes, and three and four C20‐*GA2ox* genes, respectively (Huang et al., 2015). For comparison, we also analyzed the occurrence of *GA2ox* genes in other flowering plants in our dataset. Putative *GA2ox* genes were identified in all the flowering plants analyzed except *Pogostemon cablin* and *Zostera muelleri* (Table S2). It remains to be determined whether the absence of *GA2ox* genes in *P. cablin* and *Z. muelleri* is a fact or due to the poor assembling and/or annotation of their respective genome. Consistent with the observation in Arabidopsis and rice, most plants contain more putative C19‐*GA2ox* genes than C20‐*GA2ox* genes (Table S2). The presence of *GA2ox* gene in 246 plants out of the 248 flowering plants analyzed indicate its ubiquitous occurrence, a sharp contrast to the sporadic distribution of *GAMT* gene among flowering plants.

Given the absence of *GAMT* orthologs in ~ 70% of the plant species analyzed in this study, we asked whether GAMT catalytic activity may have been maintained by SABATHs from the non‐GAMT clades. To test this possibility, we chose *Brachypodium distachyon*, a monocot in Poales, as a model species. The *B. distachyon* genome contains 12 *SABATH* genes with 10 of them being intact (Table S3). Full‐length cDNAs for all 10 intact *SABATH* genes were expressed in *E. coli*, and their respective recombinant proteins tested with GA_3_ and GA_4_. None of the 10 SABATHs proteins had methylating activity with GA_3_ or GA_4_, supporting the loss of GAMT activity in *GAMT*‐absent plants.

## 4 CONCLUSIONS AND IMPLICATIONS

By analyzing *SABATH* genes from a wide spectrum of land plants ranging from basal lineages (liverwort, moss), non‐seed vascular plants (lycophyte and ferns) to gymnosperms and angiosperms, we identified a *GAMT* clade (Figure 1) that arose early in the evolution of seed plants. In vitro enzyme assays and gene expression analysis led to two observations. We found that the catalytic activity of GAMTs for GA‐methylation (Figure 2c) and their biological function in reproduction (Figure 3) are generally conserved. The second observation is that functional divergence has also occurred, evidenced by different substrate specificity with GA_3_ and GA_4_ (Figure 2c) and by tissue‐specific expression of *GAMT* in certain species (e.g., in the senesced leaves of grapevine) (Figure 3). Such properties of GAMTs as a GA‐inactivating mechanism may have contributed to achieving lineage/developmental program‐specific functions of GAs. Equally important is the finding that *GAMT* gene is absent in approximately 2/3 of the flowering plants analyzed in this study (Figure 5). The direct consequence for the loss of *GAMT* gene is the lack of ability to inactivate GAs through methylation. While genetic innovations through gene duplication have been an engine for speciation, lineage‐specific losses of genes have also occurred frequently during eukaryote evolution (Aravind et al., 2000), including plants (Cannell et al., 2020; Gu et al., 2016). Loss‐of‐function may accompany key evolutionary transitions. For example, floral scent, which evolved early in flowering plants, has experienced repeated independent losses due to the transitions in pollinator types or modes of pollination (Raguso, 2016). It will be of great importance to determine the significance of the repeated loss of *GAMT* gene in the radiation of some of the largest lineages of flowering plants, including Poales, Fabales, and Superasterids (Figure 5). Finally, it is noteworthy that many major crops (e.g., cereal grasses and legumes) do not contain *GAMT* genes. While the lack of a *GAMT* gene may be advantageous, for certain agronomic traits (such as bushy phenotype), *GAMT* could be a useful new molecular tool for the genetic improvement of some of these *GAMT*‐lacking crops.

## 5 ACCESSION NUMBERS

The sequences for the biochemically characterized GAMT reported in this paper have been deposited in the GenBank database (accession numbers: MW149492 ‐ MW149515).

## Supporting information

Table S1‐S5
