# Supplementary material for: Origin and evolution of a gibberellin‐deactivating enzyme GAMT
Source: Plant Direct. 2020 Dec 25;4(12):e00287. doi: 10.1002/pld3.287 (PMC7762392; doi:10.1002/pld3.287)
Supplement: Supplementary file 1 — Table S1‐S5 [file PLD3-4-e00287-s001.pdf]

Table S1. **Plant genomes analyzed in this study.**

Plant species	Depository	Literature References
Actinidia chinensis	NCBI Genome	
Aegilops tauschii	Phytozome	
Aethionema arabicum	Brassica Database	
Amaranthus hypochondriacus	Phytozome	
Amborella trichopoda	Phytozome	
Ammopiptanthus nanus		(Gao et al., 2018)
Anacardium occidentale	Phytozome	
Ananas comosus	Phytozome	
Apostasia shenzhenica		(Zhang et al., 2017)
Antirrhinum majus		(Li et al., 2019)
Aquilegia coerulea	Phytozome	
Arabidopsis halleri	Phytozome	
Arabidopsis lyrata	Phytozome	
Arabidopsis thaliana	Phytozome	
Arachis duranensis		(Bertioli et al., 2015)
Arachis ipaensis		(Bertioli et al., 2015)
Arachis monticola		(Yin et al., 2018)
Artemisia annua		(Lin et al., 2017)
Artocarpus camansi		(Gardner et al., 2016)
Asparagus officinalis	Phytozome	
Atalantia buxifolia		(Wang et al., 2017)
Avicennia marina		http://evolution.sysu.edu.cn/Sequences.html
Azolla filiculoides		(Li et al., 2018)
Barbarea vulgaris		(Byrne et al., 2017)
Beta vulgaris	Phytozome	
Dorcoceras hygrometricum		(Xiao et al., 2015)
Bombax ceiba		(Gao et al., 2018)
Boechera retrofracta		(Kliver et al., 2018)
Boechera stricta	Phytozome	
Boehmeria nivea		(Luan et al., 2018)
Brachypodium distachyon	Phytozome	
Brachypodium hybridum	Phytozome	
Brachypodium stacei	Phytozome	
Brachypodium sylvaticum	Phytozome	
Brassica napus	Brassica Database	

Brassica oleracea	Brassica Database	
Brassica rapa	Brassica Database	
Cajanus cajan		(Varshney et al., 2012)
Calamus jenkinsianus		(Zhao et al., 2018)
Calamus simplicifolius		(Zhao et al., 2018)
Calotropis gigantea		(Hoopes et al., 2018)
Camelina sativa	Brassica Database	
Camellia sinensis		(Wei et al., 2018)
Camptotheca acuminata		(Zhao et al., 2017)
Cannabis sativa		(van Bakel et al., 2011)
Capsella grandiflora		(Slotte et al., 2013)
Capsella rubella	Brassica Database	
Capsicum annuum		(Kim et al., 2014)
Capsicum baccatum		(Kim et al., 2017)
Capsicum chinense		(Kim et al., 2017)
Cardamine hirsuta		(Gan et al., 2016)
Carica papaya	Phytozome	
Castanea mollissima	HWG	
Catharanthus roseus		(Kellner et al., 2015)
Cephalotus follicularis		(Fukushima et al., 2017)
Chenopodium quinoa	Phytozome	
Chrysanthemum indicum		(Song et al., 2018)
Cicer arietinum	Phytozome	
Cicer reticulatum		(Gupta et al., 2016)
Cinnamomum micranthum	NCBI Genome	
Citrullus lanatus		(Guo et al., 2013)
Citrus clementina	Phytozome	
Citrus maxima	Citrus Genome Database	
Citrus cavaleriei	Citrus Genome Database	
Citrus medica	Citrus Genome Database	
Citrus reticulata		(Wang et al., 2018)
Citrus sinensis	Phytozome	
Citrus unshiu		(Shimizu et al., 2017)
Cocos nucifera		(Xiao et al., 2017)
Coffea arabica	NCBI Genome	
Coffea canephora	Phytozome	
Corchorus capsularis	Phytozome	

Corchorus olitorius		(Sarkar et al., 2017)
Cucumis melo	CuGenDB	
Cucumis sativus	Phytozome	
Cucurbita pepo	CuGenDB	
Cynara cardunculus		(Scaglione et al., 2016)
Daucus carota	Phytozome	
Dendrobium officinale		(Yan et al., 2015)
Dianthus caryophyllus		(Yagi et al., 2013)
Dichanthelium oligosanthes		(Studer et al., 2016)
Dioscorea rotundata	Phytozome	
Durio zibethinus		(Teh et al., 2017)
Echinochloa crus-galli		(Guo et al., 2017)
Elaeis guineensis		(Jin et al., 2016)
Eragrostis tef		(Cannarozzi et al., 2014)
Erigeron breviscapus		(Yang et al., 2017)
Eschscholzia californica		(Hori et al., 2017)
Eucalyptus camaldulensis	NCBI Genome	
Eucalyptus grandis	Phytozome	
Eutrema salsugineum	Brassica Database	
Fagopyrum esculentum	NCBI Genome	
Fagopyrum tataricum	NCBI Genome	
Fagus sylvatica	NCBI Genome	
Faidherbia albida		(Chang et al., 2018)
Fragaria vesca	Phytozome	
Fraxinus excelsior	NCBI Genome	
Genlisea aurea	NCBI Genome	
Ginkgo biloba		(Guan et al., 2016)
Glycine max	Phytozome	
Glycine soja		(Qi et al., 2014)
Glycyrrhiza uralensis		(Mochida et al., 2017)
Gnetum montanum		(Wan et al., 2018)
Gossypium arboreum	NCBI Genome	
Gossypium barbadense	NCBI Genome	
Gossypium hirsutum	Phytozome	
Gossypium raimondii	Phytozome	
Handroanthus impetiginosus	NCBI Genome	
Helianthus annuus	Phytozome	

Hevea brasiliensis	NCBI Genome	
Hordeum vulgare	Phytozome	
Humulus lupulus	NCBI Genome	
Ipomoea batatas	NCBI Genome	
Ipomoea nil	NCBI Genome	
Ipomoea trifida	NCBI Genome	
Jatropha curcas	NCBI Genome	
Juglans regia	NCBI Genome	
Kalanchoe fedtschenkoi	Phytozome	
Kalanchoe laxiflora	Phytozome	
Lablab purpureus		(Chang et al., 2018)
Lactuca sativa	Phytozome	
Lagenaria siceraria	CuGenDB	
Leavenworthia alabamica	Brassica Database	
Leersia perrieri	Phytozome	
Linum usitatissimum	Phytozome	
Liriodendron chinense	NCBI Genome	
Lolium perenne	NCBI Genome	
Lotus japonicus	Phytozome	
Lupinus angustifolius	Phytozome	
Macleaya cordata	NCBI Genome	
Malania oleifera		(Xu et al., 2019)
Malus domestica	Phytozome	
Manihot esculenta	Phytozome	
Marchantia polymorpha	Phytozome	
Medicago truncatula	Phytozome	
Erythranthe guttata	NCBI Genome	
Miscanthus sinensis	Phytozome	
Momordica charantia	NCBI Genome	
Moringa oleifera		(Chang et al., 2018)
Morus notabilis	NCBI Genome	
Musa itinerans	NCBI Genome	
Musa acuminata	Phytozome	
Musa balbisiana		(Davey et al., 2013)
Musa schizocarpa	NCBI Genome	
Nelumbo nucifera	NCBI Genome	
Nicotiana attenuata	Phytozome	
Nicotiana benthamiana	NCBI Genome	
Nicotiana obtusifolia	NCBI Genome	

Nicotiana sylvestris	NCBI Genome	
Nicotiana tabacum	NCBI Genome	
Nicotiana tomentosiformis	NCBI Genome	
Ocimum tenuiflorum	NCBI Genome	
Olea europaea	Phytozome	
Oropetium thomaeum	Phytozome	
Oryza barthii	Phytozome	
Oryza brachyantha	Phytozome	
Oryza glaberrima	Phytozome	
Oryza longistaminata	Phytozome	
Oryza meridionalis	Phytozome	
Oryza sativa f. spontanea	Phytozome	
Oryza meyeriana var. granulata		(Wu et al., 2018)
Oryza punctata	Phytozome	
Oryza rufipogon	Phytozome	
Oryza sativa	Phytozome	
Panax ginseng		(Kim et al., 2018)
Panicum hallii	Phytozome	
Panicum virgatum	Phytozome	
Parasponia andersonii	NCBI Genome	
Cenchrus americanus	NCBI Genome	
Petunia axillaris		(Bombarely et al., 2016)
Petunia integrifolia subsp. inflata		(Bombarely et al., 2016)
Phalaenopsis aphrodite	NCBI Genome	
Phalaenopsis equestris	NCBI Genome	
Phaseolus vulgaris	Phytozome	
Phoenix dactylifera	NCBI Genome	
Phyllostachys edulis		(Fei et al., 2018)
Physcomitrella patens	Phytozome	
Picea abies	NCBI Genome	
Pinus lambertiana	NCBI Genome	
Pinus taeda	NCBI Genome	
Pogostemon cablin	NCBI Genome	
Populus deltoides	Phytozome	
Populus euphratica	NCBI Genome	
Populus pruinosa		(Wang et al., 2017)
Populus tremula		(Lin et al., 2018)

Populus tremuloides		(Lin et al., 2018)
Populus trichocarpa	Phytozome	
Potentilla micrantha		(Giongo et al., 2017)
Primula veris	NCBI Genome	
Primula vulgaris	NCBI Genome	
Prunus avium	NCBI Genome	
Prunus mume	NCBI Genome	
Prunus persica	Phytozome	
Pseudotsuga menziesii	NCBI Genome	
Punica granatum	NCBI Genome	
Pyrus x bretschneideri	NCBI Genome	
Pyrus communis		(Chagné et al., 2014)
Quercus lobata	NCBI Genome	
Quercus robur	NCBI Genome	
Quercus suber	NCBI Genome	
Raphanus raphanistrum	NCBI Genome	
Raphanus sativus	NCBI Genome	
Rhizophora apiculata		http://evolution.sysu.edu.cn/Sequences.html
Rhododendron delavayi		(Yan et al., 2017)
Ricinus communis	Phytozome	
Rosa chinensis	NCBI Genome	
Rosa multiflora	NCBI Genome	
Rubus occidentalis		(Wai et al., 2018)
Saccharum spontaneum	NCBI Genome	
Salix purpurea	Phytozome	
Salvia miltiorrhiza		(Zhang et al., 2015)
Salvia splendens		(Dong et al., 2018)
Salvinia cucullata		(Li et al., 2018)
Schrenkiella parvula	Brassica Database	
Sclerocarya birrea		(Chang et al., 2018)
Secale cereale	NCBI Genome	
Selaginella moellendorffii	Phytozome	
Sesamum indicum	NCBI Genome	
Setaria italica	Phytozome	
Setaria viridis	Phytozome	
Siraitia grosvenorii		(Xia et al., 2018)
Sisymbrium irio	Brassica Database	
Solanum lycopersicum	Phytozome	
Solanum melongena	NCBI Genome	

Solanum pennellii	NCBI Genome	
Solanum pimpinellifolium	NCBI Genome	
Solanum tuberosum	Phytozome	
Sonneratia alba		http://evolution.sysu.edu.cn/Sequences.html
Sonneratia caseolaris		http://evolution.sysu.edu.cn/Sequences.html
Sorghum bicolor	Phytozome	
Sphagnum fallax	Phytozome	
Spinacia oleracea	NCBI Genome	
Spirodela polyrhiza	Phytozome	
Taraxacum kok-saghyz		(Lin et al., 2017)
Tarenaya hassleriana	NCBI Genome	
Tectona grandis		(Zhao et al., 2019)
Theobroma cacao	Phytozome	
Thinopyrum intermedium	Phytozome	
Thlaspi arvense		(Dorn et al., 2015)
Trema orientale		(van Velzen et al., 2018)
Trifolium pratense	Phytozome	
Trifolium subterraneum		(Hirakawa et al., 2016)
Triticum aestivum	Phytozome	
Triticum turgidum		(Avni et al., 2017)
Triticum urartu	Phytozome	
Utricularia gibba		(Ibarra-Laclette et al., 2013)
Vaccinium corymbosum		(Gupta et al., 2015)
Vigna angularis		(Kang et al., 2015)
Vigna radiata		(Kang et al., 2014)
Vigna subterranea		(Chang et al., 2018)
Vigna unguiculata	Phytozome	
Vitis vinifera	Phytozome	
Zea mays	Phytozome	
Zizania latifolia		(Guo et al., 2015)
Ziziphus jujuba		(Liu et al., 2014)
Zostera marina	Phytozome	
Zostera muelleri		(Lee et al., 2016)
Zoysia japonica		(Tanaka et al., 2016)
Zoysia matrella		(Tanaka et al., 2016)
Zoysia pacifica		(Tanaka et al., 2016)

Table S2. Number of putative *GA2ox* genes in flowering plants.

Plant species	Total number of *GA2ox* genes	C_{19}-GA2ox number	C_{20}-GA2ox number
Actinidia chinensis	14	10	4
Aegilops tauschii	3	1	2
Aethionema arabicum	5	5	0
Amaranthus hypochondriacus	5	3	2
Amborella trichopoda	2	1	1
Ammopiptanthus nanus	10	7	3
Anacardium occidentale	16	11	5
Ananas comosus	7	4	3
Apostasia shenzhenica	7	6	1
Antirrhinum majus	13	7	6
Aquilegia coerulea	8	5	3
Arabidopsis halleri	5	4	1
Arabidopsis lyrata	8	6	2
Arabidopsis thaliana	8	5	3
Arachis duranensis	9	7	2
Arachis ipaensis	10	7	3
Arachis monticola	15	11	4
Artemisia annua	11	7	4
Artocarpus camansi	4	2	2
Asparagus officinalis	6	4	2
Atalantia buxifolia	17	5	12
Avicennia marina	12	9	3
Barbarea vulgaris	4	3	1
Beta vulgaris	3	2	1
Dorcoceras hygrometricum	5	5	0
Bombax ceiba	18	13	5
Boechera retrofracta	8	6	2
Boechera stricta	8	6	2
Boehmeria nivea	6	2	4
Brachypodium distachyon	17	13	4
Brachypodium hybridum	24	16	8
Brachypodium stacei	10	7	3
Brachypodium sylvaticum	9	6	3
Brassica napus	26	22	4
Brassica oleracea	14	10	4

Brassica rapa	14	11	3
Cajanus cajan	15	9	6
Calamus jenkinsianus	10	8	2
Calamus simplicifolius	7	6	1
Calotropis gigantea	5	4	1
Camelina sativa	18	15	3
Camellia sinensis	6	4	2
Camptotheca acuminata	13	8	5
Cannabis sativa	7	4	3
Capsella grandiflora	7	6	1
Capsella rubella	8	6	2
Capsicum annuum	11	6	5
Capsicum baccatum	8	5	3
Capsicum chinense	10	7	3
Cardamine hirsuta	7	5	2
Carica papaya	5	4	1
Castanea mollissima	8	5	3
Catharanthus roseus	4	3	1
Cephalotus follicularis	4	3	1
Chenopodium quinoa	4	3	1
Chrysanthemum indicum	10	6	4
Cicer arietinum	9	5	4
Cicer reticulatum	7	5	2
Cinnamomum micranthum	10	7	3
Citrullus lanatus	8	5	3
Citrus clementina	8	4	4
Citrus maxima	8	4	4
Citrus cavaleriei	12	5	7
Citrus medica	8	4	4
Citrus reticulata	7	4	3
Citrus sinensis	10	4	6
Citrus unshiu	9	4	5
Cocos nucifera	8	6	2
Coffea arabica	14	6	8
Coffea canephora	6	4	2
Corchorus capsularis	5	4	1
Corchorus olitorius	8	4	4
Cucumis melo	8	5	3
Cucumis sativus	6	3	3

Cucurbita pepo	15	10	5
Cynara cardunculus	13	7	6
Daucus carota	20	13	7
Dendrobium officinale	10	6	4
Dianthus caryophyllus	2	2	0
Dichanthelium oligosanthes	10	7	3
Dioscorea rotundata	5	2	3
Durio zibethinus	17	11	6
Echinochloa crus-galli	18	13	5
Elaeis guineensis	9	5	4
Eragrostis tef	5	4	1
Erigeron breviscapus	5	3	2
Eschscholzia californica	2	2	0
Eucalyptus camaldulensis	4	3	1
Eucalyptus grandis	6	4	2
Eutrema salsugineum	8	6	2
Fagopyrum esculentum	7	6	1
Fagopyrum tataricum	5	4	1
Fagus sylvatica	8	5	3
Faidherbia albida	12	8	4
Fragaria vesca	5	4	1
Fraxinus excelsior	22	18	4
Genlisea aurea	6	5	1
Glycine max	33	19	15
Glycine soja	22	13	9
Glycyrrhiza uralensis	9	7	2
Gossypium arboreum	16	11	5
Gossypium barbadense	42	27	15
Gossypium hirsutum	32	19	13
Gossypium raimondii	22	15	7
Handroanthus impetiginosus	10	7	3
Helianthus annuus	11	8	3
Hevea brasiliensis	15	8	7
Hordeum vulgare	23	19	4
Humulus lupulus	2	2	0
Ipomoea batatas	6	3	3
Ipomoea nil	11	8	3
Ipomoea trifida	6	5	1
Jatropha curcas	7	3	4

Juglans regia	18	7	11
Kalanchoe fedtschenkoi	9	6	3
Kalanchoe laxiflora	14	8	6
Lablab purpureus	10	6	4
Lactuca sativa	12	8	4
Lagenaria siceraria	7	4	3
Leavenworthia alabamica	10	9	1
Leersia perrieri	11	7	4
Linum usitatissimum	11	4	7
Liriodendron chinense	3	2	1
Lolium perenne	9	7	2
Lotus japonicus	5	4	1
Lupinus angustifolius	5	4	1
Macleaya cordata	4	2	2
Malania oleifera	6	4	2
Malus domestica	12	9	3
Manihot esculenta	12	7	5
Medicago truncatula	14	8	6
Erythranthe guttata	11	8	3
Miscanthus sinensis	19	13	6
Momordica charantia	8	4	4
Moringa oleifera	6	4	2
Morus notabilis	7	4	3
Musa itinerans	12	8	4
Musa acuminata	13	8	5
Musa balbisiana	13	8	5
Musa schizocarpa	11	8	3
Nelumbo nucifera	8	4	4
Nicotiana attenuata	8	5	3
Nicotiana benthamiana	19	14	5
Nicotiana obtusifolia	8	5	3
Nicotiana sylvestris	12	9	3
Nicotiana tabacum	19	14	5
Nicotiana tomentosiformis	11	7	4
Ocimum tenuiflorum	9	8	1
Olea europaea	19	13	6
Oropetium thomaeum	6	4	2
Oryza barthii	4	2	2
Oryza brachyantha	6	3	3

Oryza glaberrima	7	4	3
Oryza longistaminata	4	3	1
Oryza meridionalis	10	8	2
Oryza meyeriana var. granulata	10	7	3
Oryza punctata	9	6	3
Oryza rufipogon	9	6	3
Oryza sativa	9	5	4
Oryza sativa f. spontanea	10	7	3
Panax ginseng	12	6	6
Panicum hallii	11	8	3
Panicum virgatum	18	12	6
Parasponia andersonii	7	4	3
Cenchrus americanus	2	1	1
Petunia axillaris	10	8	2
Petunia integrifolia subsp. inflata	9	7	2
Phalaenopsis aphrodite	8	5	3
Phalaenopsis equestris	10	5	5
Phaseolus vulgaris	13	8	5
Phoenix dactylifera	10	7	3
Phyllostachys edulis	5	4	1
Pogostemon cablin	0	0	0
Populus deltoides	19	9	10
Populus euphratica	24	7	17
Populus pruinosa	14	6	8
Populus tremula	26	11	15
Populus tremuloides	14	9	5
Populus trichocarpa	21	10	11
Potentilla micrantha	6	4	2
Primula veris	5	3	2
Primula vulgaris	6	4	2
Prunus avium	14	5	9
Prunus mume	8	4	4
Prunus persica	12	4	8
Punica granatum	7	4	3
Pyrus x bretschneideri	20	9	11
Pyrus communis	11	7	4
Quercus lobata	8	3	5
Quercus robur	5	4	1

Quercus suber	8	4	4
Raphanus raphanistrum	11	8	3
Raphanus sativus	11	10	1
Rhizophora apiculata	9	5	4
Rhododendron delavayi	5	4	1
Ricinus communis	7	3	4
Rosa chinensis	7	4	3
Rosa multiflora	8	4	4
Rubus occidentalis	7	4	3
Saccharum spontaneum	11	7	4
Salix purpurea	16	10	6
Salvia miltiorrhiza	3	2	1
Salvia splendens	24	16	8
Schrenkiella parvula	7	6	1
Sclerocarya birrea	7	5	2
Secale cereale	4	2	2
Sesamum indicum	9	6	3
Setaria italica	18	10	8
Setaria viridis	13	11	2
Siraitia grosvenorii	9	6	3
Sisymbrium irio	5	4	1
Solanum lycopersicum	10	7	3
Solanum melongena	10	8	2
Solanum pennellii	15	10	5
Solanum pimpinellifolium	10	7	3
Solanum tuberosum	16	11	5
Sonneratia alba	14	11	3
Sonneratia caseolaris	14	11	3
Sorghum bicolor	12	9	3
Spinacia oleracea	9	2	7
Spirodela polyrhiza	5	3	2
Taraxacum kok-saghyz	3	3	0
Tarenaya hassleriana	8	5	3
Tectona grandis	12	8	4
Theobroma cacao	11	5	6
Thinopyrum intermedium	66	37	19
Thlaspi arvense	7	5	2
Trema orientale	7	4	3
Trifolium pratense	11	6	5

Trifolium subterraneum	5	5	0
Triticum aestivum	17	9	8
Triticum turgidum	33	20	13
Triticum urartu	4	1	3
Utricularia gibba	6	6	0
Vaccinium corymbosum	4	3	1
Vigna angularis	11	7	4
Vigna radiata	13	8	5
Vigna subterranea	12	7	5
Vigna unguiculata	14	9	5
Vitis vinifera	8	5	3
Zea mays	12	9	3
Zizania latifolia	10	7	3
Ziziphus jujuba	8	4	4
Zostera marina	4	3	1
Zostera muelleri	0	0	0
Zoysia japonica	10	6	4
Zoysia matrella	15	8	7
Zoysia pacifica	12	7	5

Table S3. Full-length *SABATH* genes in *Brachypodium distachyon*.

Gene name	Gene ID
BdSABATH1	*Bradi1g44620*
BdSABATH2	*Bradi1g43080*
BdSABATH3	*Bradi1g42760.1*
BdSABATH4	*Bradi2g47550*
BdSABATH5	*Bradi2g60180*
BdSABATH6	*Bradi2g60170.1*
BdSABATH7	*Bradi3g05550*
BdSABATH8	*Bradi3g57790*
BdSABATH9	*Bradi4g16077*
BdSABATH10	*Bradi4g16110*

Table S4. Selected *GA2ox* genes for HMM profile construction.

Gene Name	Class
AtGA2ox1	C19
AtGA2ox2	C19
AtGA2ox3	C19
AtGA2ox4	C19
AtGA2ox6	C19
AtGA2ox7	C20
AtGA2ox8	C20
OsGA2ox1	C19
OsGA2ox3	C19
OsGA2ox4	C19
OsGA2ox5	C20
OsGA2ox6	C20
OsGA2ox7	C19
OsGA2ox8	C19
OsGA2ox9	C20
SoGA2ox1	C19
SoGA2ox2	C19
SoGA2ox3	C20
CmGA2ox	C19
LsGA2ox1	C19
NtGA2ox1	C19
NtGA2ox3	C19
NtGA2ox5	C19
PcGA2ox1	C19
PaGA2ox1	C19
PsGA2ox1	C19
PsGA2ox2	C19

At: *Arabidopsis thaliana*; *Os*: *Oryza sativa*; *So*: *Spinacia oleracea*; *Cm*: *Cucurbita maxima*; *Ls*: *Lactuca sativa*; *Nt*: *Nicotiana sylvestris*; *Pc*: *Phaseolus coccineus*; *Pa*: *Populus alba*; *Ps*: *Pisum sativum*.

Table S5. Primers used in this study.

Gene name and ID	Primers	Sequence (5’→3’)
GbGAMT1	Gb1-F	ATGGATTGCTCGACTAGCGTGGTGTCA
(Gb_37441)	Gb1-R	CTATTTTCTGACAGCAAACACAACAATAACGCCA
GbGAMT2	Gb2-F	ATGGATGGTCCTACCATAGCTGCATC
(Gb_37750)	Gb2-R	TTATTCCTCAGCAAATCTGGTCATTACATC
BrGAMT1	Br1-F	ATGGATTCTTCTCGGAGCCTC
(Bra026440)	Br1-R	TCAAACCCTAATCGCTGAGACAG
BrGAMT2	Br2-F	ATGGAGTCACCAAGTCTTCCAGTAAC
(Bra028937)	Br2-R	TCATTCAATTCGGATGGCAGA
(BrGAMT3)	Br3-F	ATGCAAGGCGGAGACGGTGACGTCAG
(Bra010433)	Br3-R	TTAATACCAAACCCGAATCGCTGAAAC

Gb: *Ginkgo biloba*; *Br*: *Brassica rapa*.

Reference

Avni R, Nave M, Barad O, Baruch K, Twardziok SO, Gundlach H, Hale I, Mascher M, Spannagl M, Wiebe K (2017) Wild emmer genome architecture and diversity elucidate wheat evolution and domestication. Science **357:** 93-97

Bertioli DJ, Cannon SB, Froenicke L, Huang G, Farmer AD, Cannon EK, Liu X, Gao D, Clevenger J, Dash S (2015) The genome sequences of *Arachis duranensis* and *Arachis ipaensis*, the diploid ancestors of cultivated peanut. Nature Genetics **47:** 438

Bombarely A, Moser M, Amrad A, Bao M, Bapaume L, Barry CS, Bliek M, Boersma MR, Borghi L, Bruggmann R, Bucher M, D'Agostino N, Davies K, Druege U, Dudareva N, Egea-Cortines M, Delledonne M, Fernandez-Pozo N, Franken P, Grandont L, Heslop-Harrison JS, Hintzsche J, Johns M, Koes R, Lv X, Lyons E, Malla D, Martinoia E, Mattson NS, Morel P, Mueller LA, Muhlemann J, Nouri E, Passeri V, Pezzotti M, Qi Q, Reinhardt D, Rich M, Richert-Pöggeler KR, Robbins TP, Schatz MC, Schranz ME, Schuurink RC, Schwarzacher T, Spelt K, Tang H, Urbanus SL, Vandenbussche M, Vijverberg K, Villarino GH, Warner RM, Weiss J, Yue Z, Zethof J, Quattrocchio F, Sims TL, Kuhlemeier C (2016) Insight into the evolution of the Solanaceae from the parental genomes of *Petunia hybrida*. Nature Plants **2:** 16074

Byrne SL, Erthmann PØ, Agerbirk N, Bak S, Hauser TP, Nagy I, Paina C, Asp T (2017) The genome sequence of *Barbarea vulgaris* facilitates the study of ecological biochemistry. Scientific reports **7:** 40728

Cannarozzi G, Plaza-Wüthrich S, Esfeld K, Larti S, Wilson YS, Girma D, de Castro E, Chanyalew S, Blösch R, Farinelli L (2014) Genome and transcriptome sequencing identifies breeding targets in the orphan crop tef (*Eragrostis tef*). BMC genomics **15:** 581

Chagné D, Crowhurst RN, Pindo M, Thrimawithana A, Deng C, Ireland H, Fiers M, Dzierzon H, Cestaro A, Fontana P, Bianco L, Lu A, Storey R, Knäbel M, Saeed M, Montanari S, Kim YK, Nicolini D, Larger S, Stefani E, Allan AC, Bowen J, Harvey I, Johnston J, Malnoy M, Troggio M, Perchepied L, Sawyer G, Wiedow C, Won K, Viola R, Hellens RP, Brewer L, Bus VGM, Schaffer RJ, Gardiner SE, Velasco R (2014) The Draft Genome Sequence of European Pear (*Pyrus communis* L. 'Bartlett'). PLOS ONE **9:** e92644

Chang Y, Liu H, Liu M, Liao X, Sahu SK, Fu Y, Song B, Cheng S, Kariba R, Muthemba S (2018) The draft genomes of five agriculturally important African orphan crops. GigaScience **8:** giy152

Davey MW, Gudimella R, Harikrishna JA, Sin LW, Khalid N, Keulemans J (2013) "A draft *Musa balbisiana* genome sequence for molecular genetics in polyploid, inter- and intra-specific Musa hybrids". BMC Genomics **14:** 683

Dong A-X, Cui R-F, Zhao Z-N, Li Z-J, Cong R-C, Xin H-B, Liu H, Nie S, Sun Y-Q, Mao J-F, Yun Q-Z, Zhang R-G, Wang X-N, Maghuly F, Porth I (2018) High-quality assembly of the reference genome for scarlet sage, *Salvia splendens*, an economically important ornamental plant. GigaScience **7**

Dorn KM, Fankhauser JD, Wyse DL, Marks MD (2015) A draft genome of field pennycress (*Thlaspi arvense*) provides tools for the domestication of a new winter biofuel crop. DNA Research **22:** 121-131

FAIRsharing.org: HWG; Hardwood Genomics Project; DOI: https://doi.org/10.25504/FAIRsharing.srgkaf; Last edited: Jan. 8, 2019, 1:24 p.m.; Last accessed: Apr 16 2019 6:24 p.m.

Fei B, Zhao H, Xu H, Sun H, Wang J, Chen L, Li L, Wang S, Yang Y, Lou Y, Gao Z, Jiang Z, Zhang C, Chen C, Zhang H, Yang H, Gao Q, Yang W, Zhou X, Wei Y, Wang L, Wang S, Zhao S, Shi C, Liu X (2018) Chromosome-level reference genome and alternative splicing atlas of moso bamboo (*Phyllostachys edulis*). GigaScience **7**

Fukushima K, Fang X, Alvarez-Ponce D, Cai H, Carretero-Paulet L, Chen C, Chang T-H, Farr KM, Fujita T, Hiwatashi Y (2017) Genome of the pitcher plant *Cephalotus* reveals genetic changes associated with carnivory. Nature ecology & evolution **1:** 0059

Gan X, Hay A, Kwantes M, Haberer G, Hallab A, Ioio RD, Hofhuis H, Pieper B, Cartolano M, Neumann U (2016) The *Cardamine hirsuta* genome offers insight into the evolution of morphological diversity. Nature plants **2:** 16167

Gao F, Wang X, Li X, Xu M, Li H, Abla M, Sun H, Wei S, Feng J, Zhou Y (2018) Long-read sequencing and de novo genome assembly of *Ammopiptanthus nanus*, a desert shrub. GigaScience

Gao Y, Wang H, Liu C, Chu H, Dai D, Song S, Yu L, Han L, Fu Y, Tian B (2018) De novo genome assembly of the red silk cotton tree (*Bombax ceiba*). GigaScience **7:** giy051

Gardner EM, Johnson MG, Ragone D, Wickett NJ, Zerega NJ (2016) Low- coverage, whole- genome sequencing of *Artocarpus camansi* (Moraceae) for phylogenetic marker development and gene discovery. Applications in plant sciences **4:** 1600017

Giongo L, Šurbanovski N, Velasco R, Sargent DJ, Buti M, Brilli M, Cestaro A, Engelen K, Moretto M, Sonego P, Cavallini A, Barghini E, Mascagni F, Natali L, Borodovsky M, Lomsadze A, Ward JA, Alonge M, Varotto C (2017) The genome sequence and transcriptome of *Potentilla micrantha* and their comparison to *Fragaria vesca* (the woodland strawberry). GigaScience **7**

Guan R, Zhao Y, Zhang H, Fan G, Liu X, Zhou W, Shi C, Wang J, Liu W, Liang X (2016) Draft genome of the living fossil *Ginkgo biloba*. Gigascience **5:** 49

Guo L, Qiu J, Han Z, Ye Z, Chen C, Liu C, Xin X, Ye CY, Wang YY, Xie H (2015) A host plant genome (*Zizania latifolia*) after a century- long endophyte infection. The Plant Journal **83:** 600-609

Guo L, Qiu J, Ye C, Jin G, Mao L, Zhang H, Yang X, Peng Q, Wang Y, Jia L (2017) *Echinochloa crus-galli* genome analysis provides insight into its adaptation and invasiveness as a weed. Nature communications **8:** 1031

Guo S, Zhang J, Sun H, Salse J, Lucas WJ, Zhang H, Zheng Y, Mao L, Ren Y, Wang Z (2013) The draft genome of watermelon (*Citrullus lanatus*) and resequencing of 20 diverse accessions. Nature genetics **45:** 51

Gupta S, Nawaz K, Parween S, Roy R, Sahu K, Kumar Pole A, Khandal H, Srivastava R, Kumar Parida S, Chattopadhyay D (2016) Draft genome sequence of *Cicer reticulatum* L., the wild progenitor of chickpea provides a resource for agronomic trait improvement. Dna Research **24:** 1-10

Gupta V, Estrada AD, Blakley I, Reid R, Patel K, Meyer MD, Andersen SU, Brown AF, Lila MA, Loraine AE (2015) RNA-Seq analysis and annotation of a draft blueberry genome assembly identifies candidate genes involved in fruit ripening, biosynthesis of bioactive compounds, and stage-specific alternative splicing. Gigascience **4:** 5

Hirakawa H, Kaur P, Shirasawa K, Nichols P, Nagano S, Appels R, Erskine W, Isobe SN (2016) Draft genome sequence of subterranean clover, a reference for genus *Trifolium*. Scientific reports **6:** 30358

Hoopes GM, Hamilton JP, Kim J, Zhao D, Wiegert-Rininger K, Crisovan E, Buell CR (2018) Genome assembly and annotation of the medicinal plant *Calotropis gigantea*, a producer of anticancer and antimalarial cardenolides. G3: Genes, Genomes, Genetics **8:** 385-391

Hori K, Yamada Y, Purwanto R, Minakuchi Y, Toyoda A, Hirakawa H, Sato F (2017) Mining of the uncharacterized cytochrome p450 genes involved in alkaloid biosynthesis in *California poppy* using a draft genome sequence. Plant and Cell Physiology **59:** 222-233

Ibarra-Laclette E, Lyons E, Hernández-Guzmán G, Pérez-Torres CA, Carretero-Paulet L, Chang T-H, Lan T, Welch AJ, Juárez MJA, Simpson J (2013) Architecture and evolution of a minute plant genome. Nature **498:** 94

Jin J, Lee M, Bai B, Sun Y, Qu J, Alfiko Y, Lim CH, Suwanto A, Sugiharti M, Wong L (2016) Draft genome sequence of an elite *Dura* palm and whole-genome patterns of DNA variation in oil palm. DNA research **23:** 527-533

Kang YJ, Kim SK, Kim MY, Lestari P, Kim KH, Ha B-K, Jun TH, Hwang WJ, Lee T, Lee J (2014) Genome sequence of mungbean and insights into evolution within *Vigna* species. Nature communications **5:** ncomms6443

Kang YJ, Satyawan D, Shim S, Lee T, Lee J, Hwang WJ, Kim SK, Lestari P, Laosatit K, Kim KH (2015) Draft genome sequence of adzuki bean, Vigna angularis. Scientific reports **5:** 8069

Kellner F, Kim J, Clavijo BJ, Hamilton JP, Childs KL, Vaillancourt B, Cepela J, Habermann M, Steuernagel B, Clissold L (2015) Genome- guided investigation of plant natural product biosynthesis. The Plant Journal **82:** 680-692

Kim N-H, Jayakodi M, Lee S-C, Choi B-S, Jang W, Lee J, Kim HH, Waminal NE, Lakshmanan M, van Nguyen B, Lee YS, Park H-S, Koo HJ, Park JY, Perumal S, Joh HJ, Lee H, Kim J, Kim IS, Kim K, Koduru L, Kang KB, Sung SH, Yu Y, Park DS, Choi D, Seo E, Kim S, Kim Y-C, Hyun DY, Park Y-I, Kim C, Lee T-H, Kim HU, Soh MS, Lee Y, In JG, Kim H-S, Kim Y-M, Yang D-C, Wing RA, Lee D-Y, Paterson AH, Yang T-J (2018) Genome and evolution of the shade-requiring medicinal herb *Panax ginseng*. Plant Biotechnology Journal **16:** 1904-1917

Kim S, Park J, Yeom S-I, Kim Y-M, Seo E, Kim K-T, Kim M-S, Lee JM, Cheong K, Shin H-S (2017) New reference genome sequences of hot pepper reveal the massive evolution of plant disease-resistance genes by retroduplication. Genome biology **18:** 210

Kim S, Park M, Yeom S-I, Kim Y-M, Lee JM, Lee H-A, Seo E, Choi J, Cheong K, Kim K-T (2014) Genome sequence of the hot pepper provides insights into the evolution of pungency in *Capsicum* species. Nature genetics **46:** 270

Kliver S, Rayko M, Komissarov A, Bakin E, Zhernakova D, Prasad K, Rushworth C, Baskar R, Smetanin D, Schmutz J (2018) Assembly of the *Boechera retrofracta* Genome and Evolutionary Analysis of Apomixis-Associated Genes. Genes **9:** 185

Lee H, Golicz AA, Bayer P, Jiao Y, Tang H, Paterson AH, Sablok G, Krishnaraj RR, Chan C-KK, Batley J (2016) The genome of a southern hemisphere seagrass species (*Zostera muelleri*). Plant physiology**:** pp. 00868.02016

Li F-W, Brouwer P, Carretero-Paulet L, Cheng S, De Vries J, Delaux P-M, Eily A, Koppers N, Kuo L-Y, Li Z (2018) Fern genomes elucidate land plant evolution and cyanobacterial symbioses. Nature plants **4:** 460

Li M, Zhang D, Gao Q, Luo Y, Zhang H, Ma B, Chen C, Whibley A, Zhang Ye, Cao Y (2019) Genome structure and evolution of *Antirrhinum majus* L. Nature plants**:** 1

Lin T, Xu X, Cheng Z, Yu H, Li J, Xiong G, Ruan J, Huang S, Wu S, Wang X, Shao X, Qin B, Gan L, Liu S, Yang Y, Yang S, Zhang Z (2017) Genome analysis of *Taraxacum kok-saghyz* Rodin provides new insights into rubber biosynthesis. National Science Review **5:** 78-87

Lin T, Xu X, Ruan J, Liu S, Wu S, Shao X, Wang X, Gan L, Qin B, Yang Y (2017) Genome analysis of *Taraxacum kok-saghyz* Rodin provides new insights into rubber biosynthesis. National Science Review **5:** 78-87

Lin Y-C, Wang J, Delhomme N, Schiffthaler B, Sundström G, Zuccolo A, Nystedt B, Hvidsten TR, de la Torre A, Cossu RM, Hoeppner MP, Lantz H, Scofield DG, Zamani N, Johansson A, Mannapperuma C, Robinson KM, Mähler N, Leitch IJ, Pellicer J, Park E-J, van Montagu M, van de Peer Y, Grabherr M, Jansson S, Ingvarsson PK, Street NR (2018) Functional and evolutionary genomic inferences in *Populus* through genome and population sequencing of American and European aspen. Proceedings of the National Academy of Sciences **115:** E10970-E10978

Liu M-J, Zhao J, Cai Q-L, Liu G-C, Wang J-R, Zhao Z-H, Liu P, Dai L, Yan G, Wang W-J (2014) The complex jujube genome provides insights into fruit tree biology. Nature communications **5:** 5315

Luan MB, Jian JB, Chen P, Chen JH, Chen JH, Gao Q, Gao G, Zhou JH, Chen KM, Guang XM (2018) Draft genome sequence of ramie, *Boehmeria nivea* (L.) Gaudich. Molecular ecology resources **18:** 639-645

Mochida K, Sakurai T, Seki H, Yoshida T, Takahagi K, Sawai S, Uchiyama H, Muranaka T, Saito K (2017) Draft genome assembly and annotation of *Glycyrrhiza uralensis*, a medicinal legume. The Plant Journal **89:** 181-194

Qi X, Li M-W, Xie M, Liu X, Ni M, Shao G, Song C, Kay-Yuen Yim A, Tao Y, Wong F-L, Isobe S, Wong C-F, Wong K-S, Xu C, Li C, Wang Y, Guan R, Sun F, Fan G, Xiao Z, Zhou F, Phang T-H, Liu X, Tong S-W, Chan T-F, Yiu S-M, Tabata S, Wang J, Xu X, Lam H-M (2014) Identification of a novel salt tolerance gene in wild soybean by whole-genome sequencing. Nature Communications **5:** 4340

Sarkar D, Mahato AK, Satya P, Kundu A, Singh S, Jayaswal PK, Singh A, Bahadur K, Pattnaik S, Singh N (2017) The draft genome of *Corchorus olitorius* cv. JRO-524 (Navin). Genomics data **12:** 151-154

Scaglione D, Reyes-Chin-Wo S, Acquadro A, Froenicke L, Portis E, Beitel C, Tirone M, Mauro R, Monaco AL, Mauromicale G (2016) The genome sequence of the outbreeding globe artichoke constructed de novo incorporating a phase-aware low-pass sequencing strategy of F1 progeny. Scientific Reports **6:** 19427

Shimizu T, Tanizawa Y, Mochizuki T, Nagasaki H, Yoshioka T, Toyoda A, Fujiyama A, Kaminuma E, Nakamura Y (2017) Draft Sequencing of the Heterozygous Diploid Genome of Satsuma (*Citrus unshiu* Marc.) Using a Hybrid Assembly Approach. Frontiers in genetics **8:** 180

Slotte T, Hazzouri KM, Ågren JA, Koenig D, Maumus F, Guo Y-L, Steige K, Platts AE, Escobar JS, Newman LK (2013) The *Capsella rubella* genome and the genomic consequences of rapid mating system evolution. Nature genetics **45:** 831

Song C, Liu Y, Song A, Dong G, Zhao H, Sun W, Ramakrishnan S, Wang Y, Wang S, Li T (2018) The *Chrysanthemum nankingense* genome provides insights into the evolution and diversification of chrysanthemum flowers and medicinal traits. Molecular plant **11:** 1482-1491

Studer AJ, Schnable JC, Weissmann S, Kolbe AR, McKain MR, Shao Y, Cousins AB, Kellogg EA, Brutnell TP (2016) The draft genome of the C 3 panicoid grass species *Dichanthelium oligosanthes*. Genome biology **17:** 223

Tanaka H, Hirakawa H, Kosugi S, Nakayama S, Ono A, Watanabe A, Hashiguchi M, Gondo T, Ishigaki G, Muguerza M (2016) Sequencing and comparative analyses of the genomes of zoysiagrasses. DNA Research **23:** 171-180

Teh BT, Lim K, Yong CH, Ng CCY, Rao SR, Rajasegaran V, Lim WK, Ong CK, Chan K, Cheng VKY (2017) The draft genome of tropical fruit durian (*Durio zibethinus*). Nature genetics **49:** 1633

van Bakel H, Stout JM, Cote AG, Tallon CM, Sharpe AG, Hughes TR, Page JE (2011) The draft genome and transcriptome of *Cannabis sativa*. Genome biology **12:** R102

van Velzen R, Holmer R, Bu F, Rutten L, van Zeijl A, Liu W, Santuari L, Cao Q, Sharma T, Shen D (2018) Comparative genomics of the nonlegume *Parasponia* reveals insights into evolution of nitrogen-fixing rhizobium symbioses. Proceedings of the National Academy of Sciences**:** 201721395

Varshney RK, Chen W, Li Y, Bharti AK, Saxena RK, Schlueter JA, Donoghue MT, Azam S, Fan G, Whaley AM (2012) Draft genome sequence of pigeonpea (*Cajanus cajan*), an orphan legume crop of resource-poor farmers. Nature biotechnology **30:** 83

Wai CM, Colle M, Edger PP, VanBuren R, Wang J, Childs K, Liachko I, Sullivan S, Bushakra JM, Bassil NV, Vining KJ, Dossett M, Finn CE, Chagné D, Jibran R, Mockler TC (2018) A near complete, chromosome-scale assembly of the black raspberry (*Rubus occidentalis*) genome. GigaScience **7**

Wan T, Liu Z-M, Li L-F, Leitch AR, Leitch IJ, Lohaus R, Liu Z-J, Xin H-P, Gong Y-B, Liu Y, Wang W-C, Chen L-Y, Yang Y, Kelly LJ, Yang J, Huang J-L, Li Z, Liu P, Zhang L, Liu H-M, Wang H, Deng S-H, Liu M, Li J, Ma L, Liu Y, Lei Y, Xu W, Wu L-Q, Liu F, Ma Q, Yu X-R, Jiang Z, Zhang G-Q, Li S-H, Li R-Q, Zhang S-Z, Wang Q-F, van de Peer Y, Zhang J-B, Wang X-M (2018) A genome for gnetophytes and early evolution of seed plants. Nature Plants **4:** 82-89

Wang K, Yang W, Ma T, Liu J, Zhang J, Ma J (2017) The draft genome sequence of a desert tree *Populus pruinosa*. GigaScience **6**

Wang L, He F, Huang Y, He J, Yang S, Zeng J, Deng C, Jiang X, Fang Y, Wen S (2018) Genome of wild mandarin and domestication history of mandarin. Molecular Plant **11:** 1024-1037

Wang X, Xu Y, Zhang S, Cao L, Huang Y, Cheng J, Wu G, Tian S, Chen C, Liu Y (2017) Genomic analyses of primitive, wild and cultivated citrus provide insights into asexual reproduction. Nature genetics **49:** 765

Wei C, Yang H, Wang S, Zhao J, Liu C, Gao L, Xia E, Lu Y, Tai Y, She G (2018) Draft genome sequence of *Camellia sinensis* var. sinensis provides insights into the evolution of the tea genome and tea quality. Proceedings of the National Academy of Sciences**:** 201719622

Wu Z, Fang D, Yang R, Gao F, An X, Zhuo X, Li Y, Yi C, Zhang T, Liang C (2018) De novo genome assembly of *Oryza granulata* reveals rapid genome expansion and adaptive evolution. Communications biology **1:** 84

Xia M, Cheng B, Zhen G, He H, Yu R, Han X, Deng XW, Jia X (2018) Improved de novo genome assembly and analysis of the Chinese cucurbit *Siraitia grosvenorii*, also known as monk fruit or luo-han-guo. GigaScience **7**

Xiao L, Yang G, Zhang L, Yang X, Zhao S, Ji Z, Zhou Q, Hu M, Wang Y, Chen M (2015) The resurrection genome of *Boea hygrometrica*: A blueprint for survival of dehydration. Proceedings of the National Academy of Sciences**:** 201505811

Xiao Y, Xu P, Fan H, Baudouin L, Xia W, Bocs S, Xu J, Li Q, Guo A, Zhou L (2017) The genome draft of coconut (*Cocos nucifera*). GigaScience **6:** 1-11

Xu C-Q, Liu H, Zhou S-S, Zhang D-X, Zhao W, Wang S, Chen F, Sun Y-Q, Nie S, Jia K-H (2019) Genome sequence of *Malania oleifera*, a tree with great value for nervonic acid production. GigaScience

Yagi M, Kosugi S, Hirakawa H, Ohmiya A, Tanase K, Harada T, Kishimoto K, Nakayama M, Ichimura K, Onozaki T (2013) Sequence analysis of the genome of carnation (*Dianthus caryophyllus* L.). DNA Research **21:** 231-241

Yan H, Song J, Zou L, Zhang L, Ma L, Peng L, Li S, Li S, Xie W, Cai Y, Wang J, Xu P, Gao Q, Ma Y, Zhang C (2017) The draft genome assembly of *Rhododendron delavayi* Franch. var. *delavayi*. GigaScience **6**

Yan L, Wang X, Liu H, Tian Y, Lian J, Yang R, Hao S, Wang X, Yang S, Li Q (2015) The genome of *Dendrobium officinale* illuminates the biology of the important traditional Chinese orchid herb. Molecular plant **8:** 922-934

Yang J, Zhang G, Zhang J, Liu H, Chen W, Wang X, Li Y, Dong Y, Yang S (2017) Hybrid de novo genome assembly of the Chinese herbal fleabane *Erigeron breviscapus*. GigaScience **6:** 1-7

Yin D, Ji C, Ma X, Li H, Zhang W, Li S, Liu F, Zhao K, Li F, Li K (2018) Genome of an allotetraploid wild peanut *Arachis monticola*: a de novo assembly. GigaScience **7:** giy066

Zhang G, Tian Y, Zhang J, Shu L, Yang S, Wang W, Sheng J, Dong Y, Chen W (2015) Hybrid de novo genome assembly of the Chinese herbal plant danshen (*Salvia miltiorrhiza* Bunge). GigaScience **4**

Zhang G-Q, Liu K-W, Li Z, Lohaus R, Hsiao Y-Y, Niu S-C, Wang J-Y, Lin Y-C, Xu Q, Chen L-J (2017) The *Apostasia* genome and the evolution of orchids. Nature **549:** 379

Zhao D, Hamilton JP, Buell CR, Hamberger B, Johnson SR, Bhat WW, Godden GT, Soltis PS, Soltis DE, Kinser TJ, Boachon B, Dudareva N (2019) A chromosomal-scale genome assembly of *Tectona grandis* reveals the importance of tandem gene duplication and enables discovery of genes in natural product biosynthetic pathways. GigaScience **8**

Zhao D, Hamilton JP, Pham GM, Crisovan E, Wiegert-Rininger K, Vaillancourt B, DellaPenna D, Buell CR (2017) De novo genome assembly of *Camptotheca acuminata*, a natural source of the anti-cancer compound camptothecin. GigaScience **6:** 1-7

Zhao H, Wang S, Wang J, Chen C, Hao S, Chen L, Fei B, Han K, Li R, Shi C (2018) The chromosome-level genome assemblies of two rattans (*Calamus simplicifolius* and *Daemonorops jenkinsiana*). GigaScience **7:** giy097
